# [Expression of Concern] Combination of the FGFR4 inhibitor PD173074 and 5-fluorouracil reduces proliferation and promotes apoptosis in gastric cancer

**DOI:** 10.3892/or.2025.9004

**Published:** 2025-10-06

**Authors:** Yan-Wei Ye, Shuang Hu, Ying-Qiang Shi, Xie-Fu Zhang, Ye Zhou, Chun-Lin Zhao, Guo-Jun Wang, Jian-Guo Wen, Hong Zong

Oncol Rep 30: 2777–2784, 2013; DOI: 10.3892/or.2013.2796

Following the publication of this paper, it was drawn to the Editor's attention by a concerned reader that, for the western blot data shown in Figs. 1E and 4, the FGFR blots and control GAPDH blots were both strikingly similar in these figures, suggesting that the same data had been included in these, even though the results from differently performed experiments were intended to have been portrayed.

The authors were contacted by the Editorial Office to offer an explanation for these apparent anomalies in the presentation of the data in this paper; however, up to this time, no response from them has been forthcoming. Owing to the fact that the Editorial Office has been made aware of potential issues surrounding the scientific integrity of this paper, we are issuing an Expression of Concern to notify readers of this potential problem while the Editorial Office continues to investigate this matter further.

